# The relevance of the side-view in body image scales for public health: an example from two African populations

**DOI:** 10.1186/s12889-015-2511-x

**Published:** 2015-11-24

**Authors:** Emmanuel Cohen, Amadou Ndao, Gilles Boëtsch, Lamine Gueye, Patrick Pasquet, Michelle Holdsworth, Alexandre Courtiol

**Affiliations:** UMI 3189 “Environnement, Santé, Sociétés”, Faculty of Medicine–UCAD, BP 5005, Dakar-Fann, Sénégal >Africa; UMR 7206 “Ecoanthropologie et Ethnobiologie”, MNHN–CNRS, Musée de l’Homme, 17 place du Trocadéro, 75016 Paris, France; School of Health and Related Research, Public Health section, The University of Sheffield, Sheffield, Regent Court, 30 Regent Street, Sheffield, S1 4DA UK; Department of Evolutionary Genetics, Leibniz Institute for Zoo and Wildlife Research, Alfred-Kowalke-Strasse. 17, D-10315 Berlin, Germany; UMR 7206:“Ecoanthropologie et Ethnobiologie”, National Museum of Natural History, 61 rue Buffon, 75005 Paris, France

**Keywords:** Body image, Obesity, Perceptions, Body size scales, Africans

## Abstract

**Background:**

Body size scales are a common method for diagnosing body image disturbances and assessing the cultural valorisation of stoutness, a phenomenon that plays a role in the development of overweight, especially among African populations. Traditionally, body size scales present a front view. In this study, we evaluated a complementary model of representing body shape: the side view of body outlines. In particular, we examined the association between the side-view and a set of bio-anthropometric indices in men and women.

**Methods:**

To cover the inter-ethnic variability in the Niger-Congo area, we selected a balanced sex-ratio sample of 80 Cameroonians and 81 Senegalese. Individuals wearing close-fitting clothes were photographed from the front-and side-view, and measured following a bio-anthropometric protocol synthesizing body shape variation: Body Mass Index, percentage body fat, somatotype profile, waist circumference, waist-to-hip ratio, mean blood pressure and glycaemia. The shape of each front and side body outline was extracted and characterised by Normalized Elliptic Fourier Descriptors (NEFD). Finally, we assessed associations between NEFD and bio-anthropometric indices.

**Results:**

Variation in the shape of both front and side body outlines was associated with all bio-anthropometrics for at least one sex-population combination. Overall, the side view best captured body shape variation related to changes in almost all bio-anthropometrics in both sexes and populations, with the exceptions of female mesomorphy, male blood pressure and glycaemia (in both sexes). We found that the details of the relationship between bio-anthropometrics and body shape differed between the two male populations, a finding that was reflected in side-views for all criteria, but not front-views.

**Conclusions:**

Variation in body shape assessed by several bio-anthropometrics related to health and nutritional status was larger for side than front body outlines. Integrating side views in body size scales would improve the accuracy of body size assessment and thus, the assessment of behaviours leading to overweight, as well as symptoms of body image disturbances, in Africa and potentially in other populations.

**Electronic supplementary material:**

The online version of this article (doi:10.1186/s12889-015-2511-x) contains supplementary material, which is available to authorized users.

## Background

In recent decades, overweight has dramatically increased in sub-Saharan Africa due to the transition towards energy-dense diets and sedentary behaviour induced by urbanisation [1]. Being overweight has well-documented [2, 3] adverse effects on metabolic and mental health ; indeed, overweight is one of the main factors in the development of non-communicable diseases, which contribute one-third of the disability-adjusted life year burden in Africa [4]. The resulting rise in obesity is most evident in women and is increasing most rapidly amongst the poor [1].

Body image norms can influence dietary behaviour and physical activity; thus, in African populations, some studies have focussed on the impact of the traditional valorisation of stoutness on obesity prevalence [5–7]. As a consequence, some authors have shown that social preference for stoutness could be a risk factor for obesity in these populations [7, 8]. However, urbanisation has a contradictory effect: urban residents are increasingly exposed to overweight and they increase their desire for a thinner body [9]. This paradoxical effect on body image associated with urbanisation is not restricted to high income countries (HICs), but also affects many low and middle income countries (LMICs) with high rates of urbanisation [10]. Therefore, studies have focussed on body image disturbances such as eating disorders and body dysmorphic disorders, which are emerging among young urban African populations around the world [11, 12], probably as a consequence of the globalisation of the western media [13, 14].

Accurate tools for assessing body image are therefore essential for studying how body perception influences the spread of obesity and the experience of body size and shape. Due to their ease of use in the field, body size scales are a common method for measuring body image. Scales can be based on figural stimuli (drawings or silhouettes scales), photographs or computer-generated images representing individuals differing in body shape [15–18]. But whatever their basis, most body size scales rely on a front view and only a few studies have so far presented body shapes from other angles [9, 19, 20]–for example, the side-view. The potential contribution of the side-view to body image studies has already been raised [21], but no firm conclusions concerning its role in body image assessment were reached.

The general aim of our study was to determine if adding a side view representation to body scales would improve their efficiency in the assessment of body image in view of their application for public health studies**.** To do this, we assessed a large sample of African morphologies to examine quantitatively and objectively which of the two representations–front view or side view–best captures the relationship between body shape and several bio-anthropometric indices known to relate to health and/or body image assessment.

## Methods

### Scope of the study

To test the pertinence of the side view in body scales, (i) front and side photographs were taken and we simultaneously collected a series of bio-anthropometric measurements in an African sample (161 subjects). For both front and side photographs, (ii) we first digitized the silhouettes and quantified the variation in body shape of these silhouettes using a geometrical method (Normalized Elliptic Fourier Descriptors, or NEFD). The computation of NEFD allowed the characterisation of the shape of silhouettes by objective numeric values. Finally, (iii) we analysed how body shape as measured by NEFD covaried with various bio-anthropometric indices, and identified which body view (side or front) best reflected health and nutritional status (as defined by our indices).

### Sampling

We selected two large ethno-linguistic groups: Western Bantoid (Sahel region) and Bantu (equatorial forest region) which present genetic [22] and linguistic differences [23], and belonging to the language area of Africa covering the major part of sub-Saharan Africa (mainly Western, Central and Southern Africa), the Niger-Congo. These two groups differ in their macroscopic phenotypes, the first being slender and the latter more stocky [24]. Our sample therefore captured a continuum of weight gain for contrasting body morphologies and thereby represents a wide range of body shape variation among African populations. Specifically, we sampled two populations native to Western Bantoid and Bantu regions: 81 Senegalese from Dakar (31 males and 50 females) and 80 Cameroonians from Yaoundé (51 males and 29 females) during a 2-month visit in 2009.

### Producing the photographs

Subjects wore close-fitting black sportswear and were photographed after removing jewellery/distinctive objects. One front and one side (left) photograph was taken for each individual, after checking that they were standing straight with feet at 50 cm apart, arms making an angle of approximately 45° with the trunk and with palms of their open hands facing forward. All photographs were taken using a Canon 450D camera mounted on a tripod 1 m above the ground and 3.6 m away from the subject with the same 35 mm zoom (equivalent to a normal lens on a full frame camera).

### Bio-anthropometric measurements

To define the morphology of sampled individuals, the same researcher (E.C.) took a set of anthropometric measurements that were then condensed into 9 anthropometric indices. Body Mass Index (BMI, #1) was calculated by dividing body mass (in kg) by the height squared (in m^2^). The percentage of body fat (hereafter, fatness, #2) was derived from: sum of biceps, triceps, supra-iliac and subscapular skin folds [25]. The somatotype profile is a ternary composite expression of slimness index (i.e. ectomorphy, #3), musculature index (i.e. mesomorphy, #4) and fatness index (i.e. endomorphy, #5), used to characterize the morphology of subjects [26]. Body fat distribution was assessed by two proxies: waist circumference (WC, #6) and waist to hip ratio (WHR, #7), as specific measurements of abdominal obesity. Furthermore, mean blood pressure (mean BP, #8) was defined as: (diastolic BP + 1/3) x (systolic BP-diastolic BP), and a measurement of fasting blood glucose (glycaemia, #9) was taken with a glucometer. These last two indices have been shown to be associated with body weight [27], and identifying whether these physiological variables are associated with a specific view of human shape is relevant in the context of the use of body scales in the prevention of the valorisation of overweight as a risk factor for obesity [5–7].

All bio-anthropometrics were obtained following standardized procedures. Height was measured to the nearest millimetre using a portable stadiometer (SiberHegner, Switzerland). Body weight was measured in very light clothing, to the nearest 100 g, using a digital beam scale (Tanita, Japan). Computing the somatotypes was based on height and weight, tricipital, subscapular, suprasinal and medial calf skinfolds, biepicondylar humerus and femur, and arm (flexed and tensed) and calf circumferences. Circumferences were measured to the nearest millimetre, in a standing position using a non-stretchable tape measure. Skinfolds were measured using a Harpenden skinfold caliper (Holtain Ltd. UK). The biepicondylar humerus and femur bone breadths were measured using a Mitutoyodial calliper. WC was measured mid-way between the lowest rib and the iliac crest, at the end of a gentle expiration. Hip circumference was measured at the greater trochanters. Mean BP was derived for the average of two diastolic and systolic blood pressure (BP) readings, taken with the subject in a seated position, after a 15 minutes rest.

BMI, fatness, and the ternary expression of the somatotype were highly correlated (Spearman correlations, absolute rho = 0.41-0.96 for men; absolute rho = 0.64-0.95 for women). Therefore, we performed Principal Component Analysis (PCA) on these metrics (unscaled and centred) for each sex, extracting the first principal component (PC) which captured 87 % and 89 % of the total variation in men and women, respectively. This PC was then used as a synthetic measure of the overall body size defined as *corpulence*, our bio-anthropometric #10. PCA as well as all other analyses were performed, unless stated otherwise, using the R (version 3.1) statistical software.

### Body shape analysis

To assess and visualize the influence of all 10 bio-anthropometric indices on body shape, we used the elliptic Fourier shape analysis [28], which has been shown to be reliable for the study of variation in human body shape [18]. Briefly, during this analysis the outline of the body is expressed as *x* and *y* Cartesian coordinates and characterised by a function of the curvilinear abscissa representing the net distance on the outline from an arbitrary starting point [18, 28]. As such, the body outline corresponds to a periodic signal which can be approximated by a sum of trigonometric functions following traditional Fourier series expansions.

To conduct this Fourier analysis, we first delimited body outlines for each original photograph using a virtual paintbrush using Adobe Photoshop CS. We then removed hands and hair from outlines in order to suppress variation that was outside the scope of the present study. We also removed arms from side views when they overlapped with the body outline. All photographs were then transformed into the portable anymap file format (*.pnm) and loaded in R using functions from the pixmap package [29]. Outlines obtained were digitised and we computed NEFD associated with each outline [18]. NEFD are a set of coefficients that parametrise the trigonometric functions used to approximate body outlines. For all pictures, we consider a number of Fourier harmonics corresponding to a perfect pixel approximation for the shortest outline (between 3208 and 4648 depending on the view orientation and sex).

Second, we studied the influence of all 10 bio-anthropometrics on body shape, by reducing the dimensionality of the shape information by applying a PCA on the NEFD obtained for each combination of sex and view (discarding the three coefficients of the first harmonic that are constrained by normalization) [28]. We retained the first 8 principal components from each PCA. Together, they captured around 95 % of the total body shape variation for front and 99 % for side outlines for each sex. Then, we performed a multivariate regression for each bio-anthropometric index, sex and view (Additional file 1: Table S1, Additional file 2: Table S2, Additional file 3: Table S3 and Additional file 4: Table S4). In all regressions, the 8 PCs associated with NEFD defined the multivariate response and the independent variables considered were: the bio-anthropometric, height and population. For the analysis of front outlines, we also considered the angles of arms and legs (measured using imageJ software) as covariates so that variation in body shape caused by posture differences did not impede analysis.

In order to quantify the extent to which the bio-anthropometrics influence body shape, we measured the coefficient of variation in perimeter-area ratio (PAR) associated with a change in four standard deviations (mean + 2SD vs. mean-2SD) for each bio-anthropometric index. The PAR is a simple metric proposed to relate to body image perception and known to strongly correlate with different body shape measures [18]. For example, Courtiol et al. [18] showed that the PAR is strongly negatively correlated to BMI in a sample mainly constituted of Caucasians. To measure this metric, we predicted the corresponding NEFD values using the regression models refitted on all NEFD. We then reconstructed the outlines corresponding to these predictions and measured their PAR.

### Ethics

The study was approved by the Institutional Ethics Committee of the Institute of Medical Research and Medicinal Plant Studies of Cameroon. Oral consent was obtained from subjects, after they were fully informed about the study goals and methods.

## Results

### Variation in bio-anthropometrics

We found pronounced differences both between sexes and between the two ethnic groups we sampled (Table 1). In both populations, women were significantly shorter and fatter than men (lower fatness, lower ectomorphy, higher endomorphy, and a tendency towards a higher BMI). Cameroonian women were taller and tended to be more mesomorphic (i.e. more muscular) than their Senegalese counterparts. There was also differences in bio-anthropometrics between populations in men, as Cameroonians were significantly taller, and presented a higher BMI, lower ectomorphy and higher mesomorphy than Senegalese men. Cameroonian men also had a higher mean blood pressure. The synthetic index of corpulence did not differ across populations (Table 1) and should not be compared between sexes as it was created for each sex independently.

**Table 1:** Bio-anthropometric characteristics of photographed individuals

Bio-anthropometrics	Cameroonian			Senegalese			Differences between populations	
	Males (*n* = 51)	Females (*n* = 29)	*P*-values	Males (*n* = 31)	Females (*n* = 50)	*P*-values	Males (*P*-values)	Females (*P*-values)
Age	39.5 ± 14.2	35.3 ± 12.0	0.18	37.5 ± 16.0	39.9 ± 13.4	0.31	0.47	0.15
Height (cm)	170.8 ± 6.2	160.4 ± 6.3	<0.001	176.9 ± 6.2	165.0 ± 5.4	<0.001	<0.001	0.001
Body mass (kg)	77.6 ± 15.0	75.4 ± 16.4	0.54	71.9 ± 15.0	72.7 ± 16.4	0.72	0.23	0.98
BMI (kg/m^**2**^)^a^	26.5 ± 5.1	28.9 ± 6.4	0.068	23 ± 3.4	26.9 ± 6.4	0.0043	<0.001	0.29
Fatness (%)	20.1 ± 7.4	33.4 ± 6.5	<0.001	18.3 ± 8.2	33.7 ± 7.6	<0.001	0.24	0.77
Ectomorphy	1.70 ± 1.30	0.91 ± 1.10	0.002	3.19 ± 1.67	1.66 ± 1.83	<0.001	<0.001	0.079
Mesomorphy	5.78 ± 1.47	5.69 ± 1.96	0.74	4.39 ± 1.21	4.88 ± 1.83	0.23	<0.001	0.054
Endomorphy	3.30 ± 1.59	5.53 ± 1.92	<0.001	2.66 ± 1.21	5.52 ± 1.86	<0.001	0.077	0.90
WC (cm)^a^	90.6 ± 14.1	94.0 ± 15.2	0.18	80.8 ± 9.6	93 ± 17.2	<0.001	<0.001	0.93
WHR^a^	0.91 ± 0.06	0.86 ± 0.09	0.011	0.85 ± 0.06	0.88 ± 0.10	0.15	<0.001	0.56
Corpulence^b^	1.41 ± 8.75	−0.80 ± 8.80	NA	−2.32 ± 8.58	0.46 ± 10.0	NA	0.11	0.87
Mean BP (mm Hg)^a^	98.0 ± 13.8	91.0 ± 8.4	0.0011	85.3 ± 14.0	93.0 ± 16.5	0.019	<0.001	0.84
Glycaemia (mg/dl)	99.5 ± 28.8	102.8 ± 21.3	0.35	105.1 ± 21.6	113.2 ± 22.3	0.057	0.73	0.20

^a^ *BMI* body mass index, *WC* waist circumference, *WHR* waist to hip ratio, *BP* blood pressure

^b^Note that the *corpulence* metric is a synthetic measure derived for others: For men, corpulence *=* 0.47BMI + 0.85fatness-0.14ectomorphy + 0.10mesomorphy + 0.16endomorphy

For women, corpulence *=* 0.63BMI + 0.72fatness-0.15ectomorphy + 0.16mesomorphy + 0.18endomorphy. Within each sex, all variables were centred during this computation (i.e. difference to the mean value). Therefore, comparisons between sexes are meaningless and indicated as Not Applicable (NA) for this variable

Mean +/− 1 Standard Deviation (SD) are reported as well as *P*-values of Mann–Whitney *U* test comparing the bio-anthropometrics between sexes and populations. High values for ectomorphy, mesomorphy and endomorphy corresponds to having a thin body built, a muscular body and a heavy body build, respectively

### Variation in body shape

**Fig. 1:**
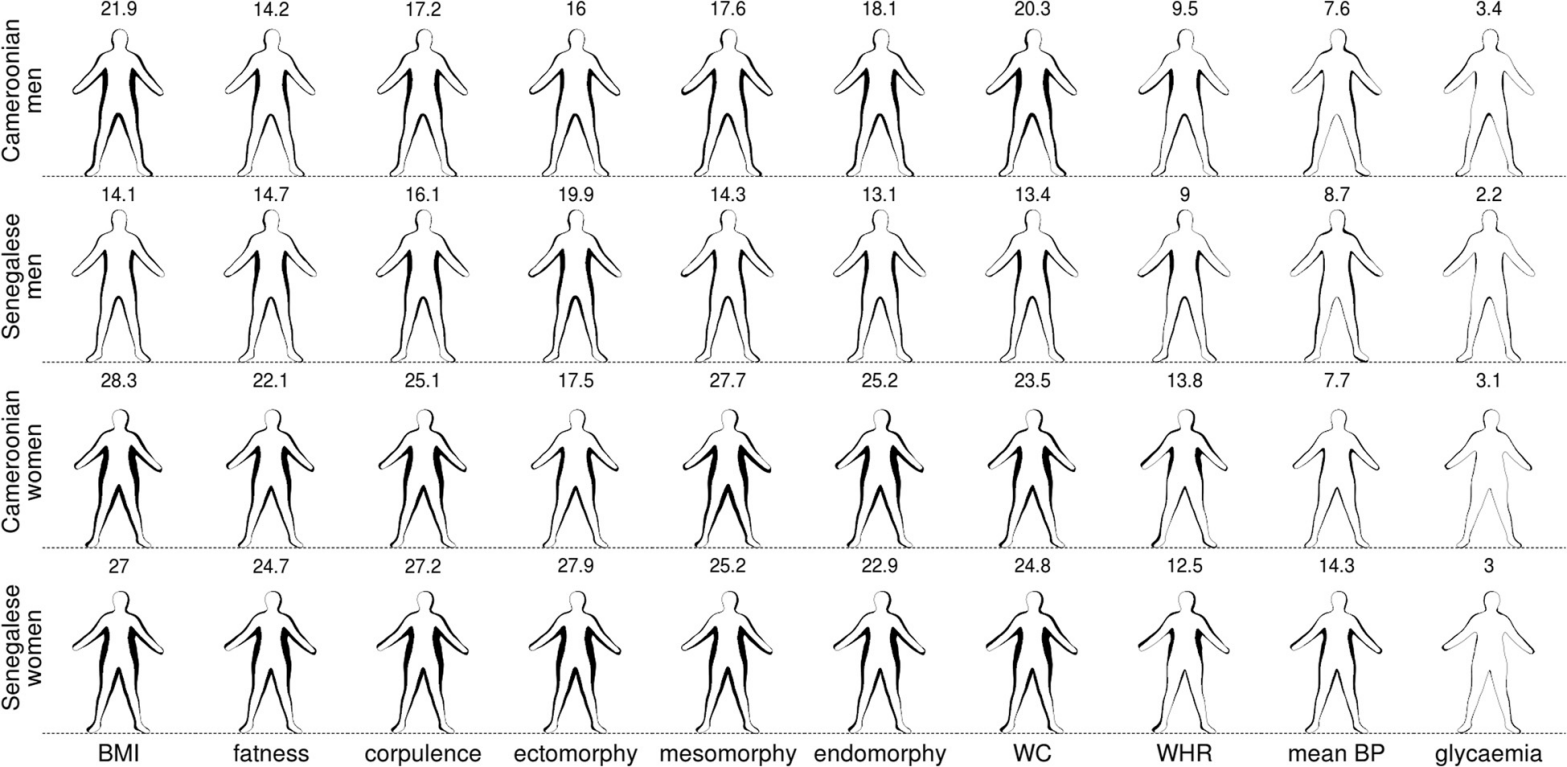
The relationship between several bio-anthropometric measures and front-view body shape. For each sex-population combination (in rows), the relationship of the different bio-anthropometrics is displayed (in columns) by superimposing the predicted outline corresponding to 2 Standard Deviations (SD) below the mean value for this bio-anthropometric with one corresponding to 2SD above the mean. Height is set to the mean value. All means and SD are computed within each sex-population combination. Differences between pairs of predicted outlines are coloured in black. The larger outline for each pair corresponds to higher bio-anthropometric values, except for ectomorphy. The coefficient of variation (%) associated with the change in perimeter-area ratio between outline pairs is indicated on top of each figurine

**Fig. 2:**
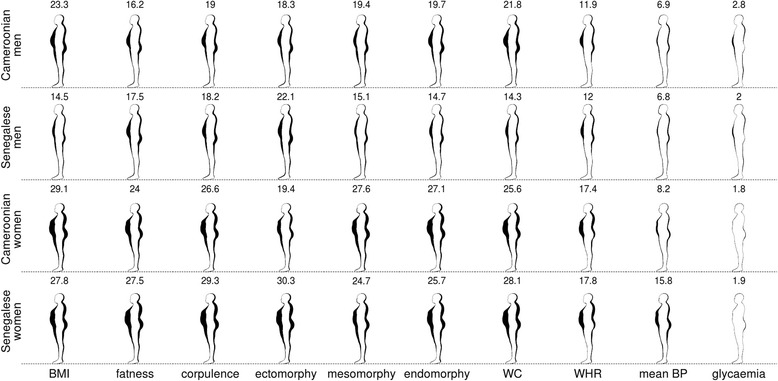
Relationship between several bio-anthropometric measures and side-view body shape. See Fig. 1 for legend

Figures 1 and 2 show how bio-anthropometrics related to body shape variation from front-and side-views, respectively. All bio-anthropometrics were significantly related to variation in body shape, with the exceptions of glycaemia for front-views in both sexes and for side-views in women, and of mean BP for side-views in men (Additional file 1: Table S1, Additional file 2: Table S2, Additional file 3: Table S3 and Additional file 4: Table S4). After accounting for the statistical effect of bio-anthropometrics, the residual variation in body shape differed significantly between populations for men but not for women (Additional file 1: Table S1, Additional file 2: Table S2, Additional file 3: Table S3 and Additional file 4: Table S4). This effect of the population on body shape appeared in all models predicting male side-views (Additional file 3: Table S3). In contrast, when the population was also considered, only regressions predicting the association between body shape of men viewed from front and changes in fatness, corpulence and endomorphy were significantly better (Additional file 1: Table S1).

Visual comparison of Figs. 1 and 2 as well as PAR analyses showed that side-views best captured body shape variation related to changes in most bio-anthropometrics for both sexes and both populations. The exceptions were mean BP for men and glycaemia for both sexes. Nonetheless, these two bio-anthropometrics were less related to body shape than any other metrics, irrespective of the view-orientation. Female mesomorphy also translated into a smaller change in PAR for side-than for front-views, but the decrease was negligible in this case.

## Discussion

In a sample based on wide range of African morphologies relevant to the Niger-Congo region, we quantified body shape variation between individuals using Fourier analysis and related it to variation in 10 different bio-anthropometrics. Our overall goal was to improve the methods for body image assessment in public health. We were particularly aiming to evaluate and compare the information conveyed by front- and side-views of body shape, to determine the potential pertinence of the side view in body size scales.

We found that body shape covaried both for front and side views with changes in bio-anthropometrics**.** Among the different bio-anthropometrics we used, all except mean BP and glycaemia were reliable predictors of body shape variation, and had different effects depending on sex and population. For example, whilst BMI reflected similarly well an increase in corpulence for women from Cameroon and Senegal, it had less connection to body shape for Senegalese men compared with Cameroonians. The finding that mean BP and glycaemia related least to body shape is unsurprising as, contrary to our other measurements, these metrics did not correspond to direct morphological measurements. Nonetheless, mean BP correlated with body shape for front-views in both sexes and for side-views in women, a finding consistent with the documented association between abdominal obesity and blood pressure [30], especially in Senegalese women. Similarly, glycaemia was significantly related to body shape changes in men viewed from the side and was weakly associated with increased abdominal obesity.

By comparing variation in body shape between front- and side-views, we found that for all the morphological criteria used to characterise individuals, the variation in body shape was larger for side- than for front-views. For the physiological criteria we considered, results are less conclusive, with blood pressure being more related to body shape variation from the front, while a significant effect of glycaemia was only noticed for side views. As these criteria are only weakly connected to body shape variation irrespectively of the representation, further investigation using larger samples will be necessary to assess the generality of these findings. The side-view was also the orientation that best reflected the body shape variation related to ethno-linguistic groups. That side view better encompasses variation in body shape may be explained by the fact that such representation captures better the morbid overweight expression on abdominal obesity. From the front, the covariation between abdominal obesity (WC and WHR) and body shape is reduced, especially when only the outline is being considered, as in the present study.

While these results support the use of side views in body scales, we think it is important to maintain the front view representation. First, when scales are based on photographs, viewers can perceive information on abdominal obesity other than its effect on the outline. Second, we also found that while the side-view reflects morphological criteria more effectively, the front-view also captures significantly body shape variation related to all morphological criteria. The side-view is thus better, but the front view is by no means useless. Third, some traits may in fact be more related to variation in body shape perceived from the front than from the side. Therefore both views contain information about the traits underlying body shape and if part of the information may be redundant between both views, it is also likely that some information can be better extracted from each angle.

Hence, we recommend the use of the two views in the design of body scales as an exhaustive model of the representation of body shape variations. Our results show that adding side views to existing African body scales representing front views only [17, 31, 32] would be an improvement. To date, drawings or human models in scales are globally represented from the front [16, 33, 34], sometimes three-quarters [19, 35] or in profile [36], but rarely from both front and profile. (A notable exception is the scale developed by Bush et al. [9] which contains the two complementary views, but this latter was used only once in Africa [37].) We are unaware of any similar study for Caucasians. Testing the pertinence of the side-view in Caucasians would test the utility of including a side view variation of body shape in Caucasians body scales. Indeed, we predict that other human populations than those investigated here would also benefit from the use of both front and side views because abdominal obesity is the most prevalent manifestation of metabolic syndrome in many populations [38].

We established in this study that there is substantial geometrical information both in front and side views: body shape traits can be used to reveal relevant traits for public health. Whether humans perceive and use such information is another question. Empirical studies of the perception of body image that simultaneously use both front and side views are therefore needed to assess the practical validity of both representations in Africa, as well as in other populations. In particular, we recall that the future use of the complementary views representation model does not exempt researchers in the field from separately validating their body scales in a representative sample of their population of interest. Local variation in body image perception and in body shapes exist [39, 40] and no method and nobody scale should be considered as being universal.

## Conclusions

Body scales are a widespread tool for studying body image and body size norms–traits known to influence a number of health outcomes. For example, body scales are used to study the social valorisation of corpulence, which could be a key determinant of obesity in Africa [5–7, 37]. But while it is clear that designing effective body scales is essential, rigorous studies quantifying what body scales actually represent are scarce. This is true in general and especially in Africa since anthropometric knowledge used during the conception of such body size scales mainly comes from studies in HICs [9, 16, 34]. Here, we assess the role of front and side representation in body scales built using African individuals. We show that the side representation provides a better and more accurate expression of the body shape variation on body size scales, probably because it encompasses the effect of progressive weight gain, especially the abdominal obesity variation.

The integration of the side view as a complement to the conventional front-view in the development of body size scales should improve our understanding of the assessment of the link between the perception of body shape variation and the spread of obesity, in Africa and potentially in other populations too. Public health concerns other than obesity may also benefit from such a methodological improvement. For example, it should improve the qualitative analysis on the mechanisms of bodily depreciation in the development of eating disorders and body dysmorphic disorders, which continue to increase in all developmental contexts [14, 41]. In sum, we hope that our study will stimulate the development and evaluation of new body scales, which once designed properly can become an effective research tool in public health.

## Additional files

Additional file 1: Table S1. Summary table of the models testing the relationship between bio-anthropometrics and front-views of men’s body shapes. Table provide the Pillai’s trace statistic and associated F-tests from multivariate analyses of variance (MANOVA) performed on the first eight principal components of the Principal Component Analysis (PCA) on Fourier coefficients (see main text for details). Analyses were performed using the function *Manova* from the R package *car*. (XLSX 11 kb) Additional file 2: Table S2. Summary table of the models testing the relationship between bio-anthropometrics and front-views of women body shapes. See Table 1 in main text. (XLSX 11 kb) Additional file 3: Table S3. Summary table of the models testing the relationship between bio-anthropometrics and side-views of men body shapes. See Table 1 in main text. (XLSX 6 kb) Additional file 4: Table S4. Summary table of the models testing the relationship between bio-anthropometrics and side-views of women body shapes. See Table 1 in main text. (XLSX 6 kb)
